# Incremental value of left atrial strain to predict atrial fibrillation recurrence after cryoballoon ablation

**DOI:** 10.1371/journal.pone.0259999

**Published:** 2021-11-19

**Authors:** Andreea Motoc, Maria–Luiza Luchian, Esther Scheirlynck, Bram Roosens, Hadischat Chameleva, Maxim Gevers, Xavier Galloo, Berlinde von Kemp, Robbert Ramak, Juan Sieira, Carlo de Asmundis, Gian–Battista Chierchia, Julien Magne, Caroline Weytjens, Steven Droogmans, Bernard Cosyns

**Affiliations:** 1 Department of Cardiology, (Centrum voor Hart- en Vaatziekten), Vrije Universiteit Brussel (VUB), Universitair Ziekenhuis Brussel, Brussels, Belgium; 2 Faculty of Medicine and Pharmacy, Vrije Universiteit Brussel, Brussels, Belgium; 3 Heart Rhythm Management Centre, Vrije Universiteit Brussel (VUB), Universitair Ziekenhuis Brussel, Brussels, Belgium; 4 Service Cardiologie, CHU Limoges, Hôpital Dupuytren, Limoges, France; 5 16 INSERM 1094, Faculté de Médecine de Limoges, Limoges, France; Policlinico Casilino, ITALY

## Abstract

**Objective:**

Atrial fibrillation (AF) recurrence occurs in approximately 25% of the patients undergoing cryoballoon ablation (CBA), leading to repeated ablations and complications. Left atrial (LA) dilation has been proposed as a predictor of AF recurrence. However, LA strain is a surrogate marker of LA mechanical dysfunction, which might appear before the enlargement of the LA. The purpose of this study was to evaluate the additional predictive value of LA function assessed using strain echocardiography for AF recurrence after CBA.

**Methods:**

172 consecutive patients (62.2 ± 12.2 years, 61% male) were prospectively analyzed. Echocardiography was performed before CBA. Blanking period was defined as the first three months post-ablation. The primary endpoint was AF recurrence after the blanking period.

**Results:**

50 (29%) patients had AF recurrence. In the overall study population, peak atrial longitudinal strain (PALS) ≤ 17% had the highest incremental predictive value for AF recurrence (HR = 9.45, 95%CI: 3.17–28.13, p < 0.001). In patients with non-dilated LA, PALS≤17% remained an independent predictor of AF recurrence (HR = 5.39, 95%CI: 1.66–17.52, p = 0.005).

**Conclusions:**

This study showed that LA function assessed by PALS provided an additional predictive value for AF recurrence after CBA, over LA enlargement. In patients with non—dilated LA, PALS also predicted AF recurrence. These findings emphasize the added value of LA strain, suggesting that it should be implemented in the systematic evaluation of AF patients before CBA.

## Introduction

Catheter ablation therapy has become a cornerstone in the treatment of symptomatic paroxysmal or persistent atrial fibrillation (AF) [1]. Although cryoballoon ablation (CBA) has shown increased safety and efficiency among several ablation techniques, AF recurrence rate after CBA remains as high as 25–30%, leading to increased rates of complications and repeated procedures [2, 3]. Consequently, the prediction of AF recurrence risk before the index ablation might allow a better patient selection, an individualized therapeutic strategy and a targeted follow—up.

Recent data have shown that left atrium (LA) structural remodeling, defined as LA dilation, predicts AF recurrence after CBA [2, 4, 5]. However, a growing body of evidence suggests that LA functional assessment might be a more sensitive prognostic indicator of AF recurrence [6–8]. LA dysfunction caused by the deposition of collagen fibers in the interstitium leading to LA fibrosis is typical in AF and might even be present in patients without LA enlargement [9].

LA strain has been proposed as a highly sensitive surrogate marker of LA structural abnormalities such as fibrosis, being able to detect the LA mechanical dysfunction at an early stage [10]. However, most of the studies were performed on small, heterogeneous populations and addressed radiofrequency ablation [11]. The early recognition of LA dysfunction might play a key role in identifying patients at higher risk of AF recurrence after CBA, even between those without LA enlargement.

In this study, we hypothesized that LA function assessed using LA strain may have additional value in the prediction of AF recurrence after CBA compared to LA size.

## Materials and methods

Two hundred and six patients diagnosed with AF (paroxysmal/persistent) and undergoing CBA as index ablation at the University Hospital of Brussels, Belgium were prospectively included from December 2018 to December 2019. Paroxysmal/persistent AF were defined according to current guidelines [1]. Exclusion criteria were: inability to sign informed consent, suboptimal image quality, severe valve disease, previous mitral surgery. Image quality was assessed according to number of visible segments and the degree of endocardial border delineation. Thirty-four patients were excluded due to suboptimal image quality.

The study was approved by the local Ethical Committee and was carried out in accordance with the ethical principles for medical research involving human subjects established by Helsinki Declaration, protecting the privacy of all participants, as well as the confidentiality of their personal information. Written informed consent was obtained from all patients.

### Transthoracic echocardiography (TTE)

All patients underwent a comprehensive TTE (GE, Vivid E95, Vingmed Ultrasound, Horten, Norway) two weeks before ablation. M-Mode, two—dimensional (2D) and Doppler measurements were performed following standard recommendations [12–14] using Echopac version 203, GE.

Left atrium anterior–posterior diameter (LAD) was measured using M-Mode, in parasternal long-axis view, perpendicular to the aortic root long axis using the leading—edge to leading—edge convention at end—systole. LA end—systolic volume (LAV) was measured using the biplane area—length method in apical four—and two—chamber views. LAD and LAV were indexed based on the patient’s body surface area.

**Fig 1:**
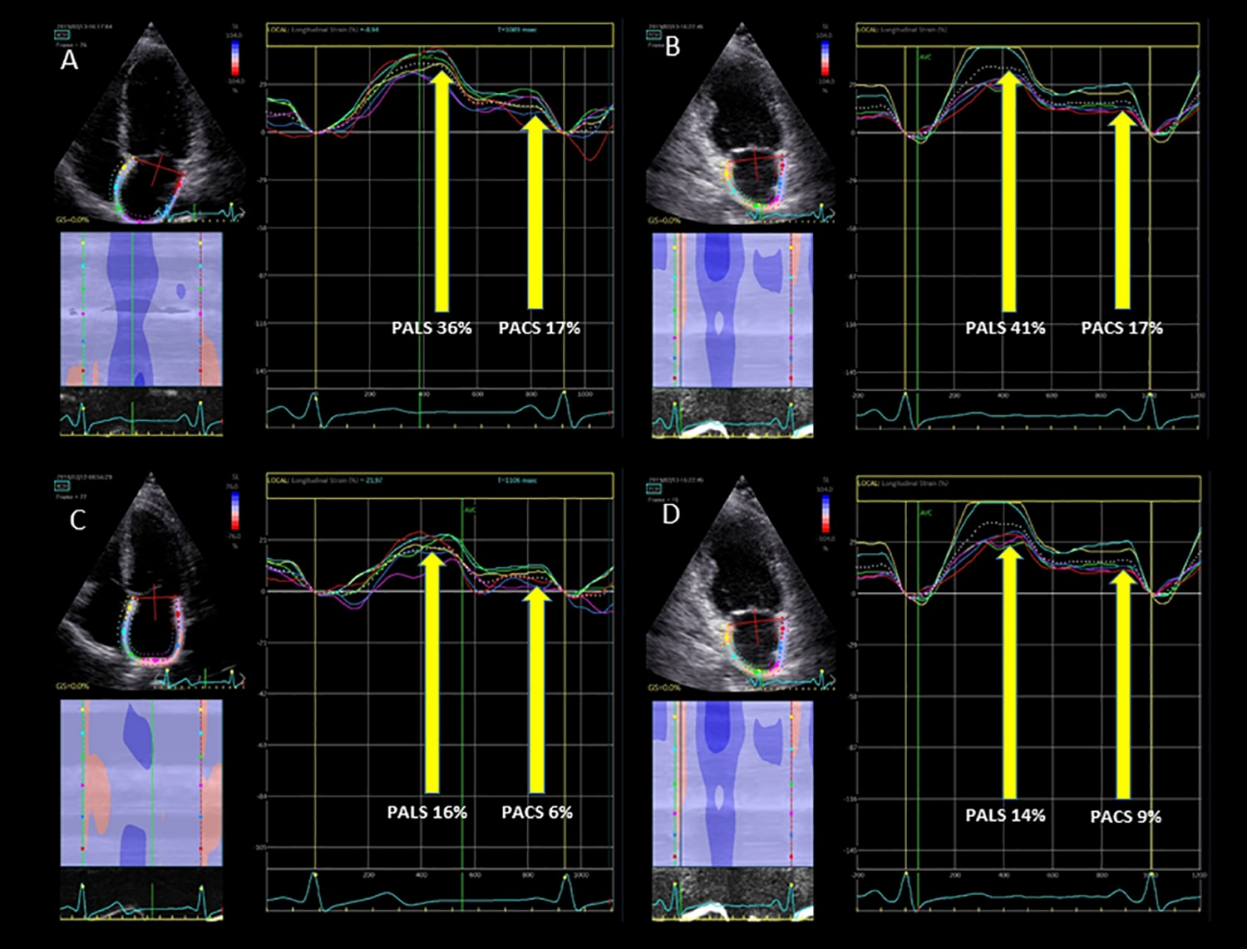
Peak atrial longitudinal strain (PALS) and peak atrial contraction strain (PACS) in a patient without atrial fibrillation recurrence (A, B) and with atrial fibrillation recurrence (C, D), respectively.

For speckle tracking, 2D grey–scale four and two- apical chamber view images were acquired with a frame rate of 50–70 frames per second. LA strain was assessed using the QRS reference method [15]. LA endocardial border was identified at end-systole in apical views and the software automatically generated a region of interest (ROI) with six segments per view. The ROI was manually adjusted if necessary. Peak atrial longitudinal strain (PALS) was measured at the level of the first, taller peak of the LA strain curve and peak atrial contraction strain (PACS) was measured at the level of the second, smaller peak of the curve (Fig 1). From the mean of the 12 segment values, global PALS and PACS were obtained. Global conduit strain was calculated by the difference between PALS and PACS. According to current recommendations, for patients in AF during the echocardiography, only PALS was measured [16].

Measurements were repeated by the same observer and by an additional investigator one month later, respectively, to assess the intra- and inter-observer reproducibility for LA parameters. All echocardiographic measurements were performed blinded to any clinical outcome.

### Pre-procedural management

Antiarrhythmic drugs (AADs) were discontinued at least 3 days before the procedure, except for amiodarone, which was stopped one month before.

### Cryoballoon ablation

After obtaining LA access, through a steerable 15 Fr sheath (FlexCath Advance®, Medtronic©), an inner lumen mapping catheter (ILMC) (Achieve®, Medtronic©) was advanced in each pulmonary vein (PV) ostium and baseline electrical information was gathered. Optimal vessel occlusion was considered as achieved upon selective contrast injection showing total contrast retention with no backflow into the atrium. Once occlusion was documented, cryothermal energy was started for at least 180 seconds. In cases of phrenic nerve palsy (PNP), recovery of diaphragmatic contraction was carefully monitored for 30 min. No further additional cryoenergy applications were applied if the veins were isolated after the initial freeze. During the whole procedure, activated clotting time was maintained > 250 seconds. In order to avoid phrenic nerve palsy, diaphragmatic stimulation was achieved by pacing the phrenic nerve during septal pulmonary veins ablations.

### Postablation management

Following the procedure, all patients were continuously monitored with electrocardiogram telemetry for at least 18 hours. Patients were discharged the day after ablation if the clinical status was stable. Oral anticoagulation therapy (OAT) was not discontinued before the ablation. Following current guidelines recommendations, OAT was continued for at least 2 months after the ablation depending on the CHA2DS2-VASc score [1]. Blanking period (BP) was defined as the first three months post-ablation. During the BP, AADs were continued. The decision to continue AADs after the BP or to repeat the procedure was taken whether AF recurrence occurred.

### Follow–Up

All patients underwent clinical follow–up and 24 hours Holter at 1, 3, 6 and 12 months after ablation. Additional Holter monitoring was performed if arrhythmic symptoms occurred. All documented AF episodes > 30 seconds were considered a recurrence.

The primary endpoint of the study was defined as AF recurrence after the BP.

### Statistical analysis

Statistical analyses were performed using IBM SPSS Statistic for Windows, Version 27.0 (Armonk, NY: IBM Corp.) and R software (version 1.4.1106, R Foundation for Statistical Computing, Vienna, Austria). Normal distribution of continuous quantitative variables was assessed by Kolmogorov—Smirnov test. Continuous variables were presented as means with standard deviations (SD) or median [with interquartile (IQR)] for skewed variables. Categorical variables were presented as numbers with percentages. Comparisons of continuous variables were done with a Student t-test or Mann-Whitney U-test and binomial variables with a Chi-squared or Fisher Exact test as appropriate. Receiver-operator characteristic (ROC) curves were constructed to evaluate the performance of echocardiographic variables in predicting AF recurrence and to calculate optimal cut-off values, specificity and sensitivity of the parameters (using Youden’s Index) in the prediction of AF recurrence. In order to evaluate potential predictors of AF recurrence, univariable and multivariable Cox regression analysis were performed to construct a baseline predictive model. The ability of LA parameters to predict AF recurrence was evaluated by comparing the additional percentage of increase of the chi-square value of combined models over the baseline model. Models were also compared using C—statistic. Kaplan—Meier survival curves were used to evaluate AF—free survival rate, and comparison between groups were performed using the log—rank test. Variables included in the statistical models were tested for collinearity using linear regression analysis, with a variance inflation factor between 1 and 10. Intraclass correlation coefficient (ICC) was used to determine intra- and interobserver reproducibility for LA measurements. Statistical significance was considered for a p–value < 0.05.

## Results

### Baseline characteristics of the study population

One hundred seventy two patients were included in the analysis (62.6 ± 12.2 years old, 61% male). Baseline clinical and demographic characteristics of the study population are shown in Table 1. Echocardiography characteristics are summarized in Table 2.

**Table 1:** Baseline characteristics of the study population.

	Total (n = 172)	No AF recurrence (n = 122) (71%)	AF recurrence (n = 50) (29%)	p
Age, years	62.6 ± 12.2	62.4 ± 11.9	63.0 ± 13.2	0.770
Male, n (%)	105 (61)	74 (60.7)	31 (62)	0.870
BMI, kg/m^2^	27.4 ± 4.8	27.3 ± 4.6	27.8 ± 5.1	0.594
Systolic blood pressure, mmHg	130.2 ± 20.7	129.7 ± 20.8	131.7 ± 20.5	0.558
Diastolic blood pressure, mmHg	75.9 ± 12.6	75.2 ± 12.6	77.8 ± 12.7	0.229
Heart rate, bpm	71.9 ±14.7	70.6 ± 14.8	75.2 ± 14.1	0.072
AF during echo, n (%)	41 (23.8)	20 (16.3)	21 (42.0)	0.001
Persistent AF, n (%)	28 (16.3)	14 (11.6)	14 (28.0)	0.008
Smoking, n (%)	18 (10.5)	10 (8.2)	8 (16.3)	0.117
Hypertension, n (%)	103 (59.9)	75 (62.0)	28 (57.1)	0.559
Diabetes mellitus, n (%)	18 (10.5)	15 (12.4)	3 (6.0)	0.215
Dyslipidemia, n (%)	97 (56.4)	72 (59.0)	25 (51.0)	0.340
CAD, n (%)	34 (19.8)	26 (21.7)	8 (16.7)	0.466
CVA/TIA, n (%)	9 (5.2)	7 (5.7)	2 (4.0)	0.642
Heart failure, n (%)	14 (8.1)	7 (5.8)	7 (14.3)	0.070
Valvular disease, n (%)	6 (3.5)	2 (2.6)	4 (11.1)	0.060
COPD/asthma, n (%)	12 (7.0)	9 (7.4)	3 (6.0)	0.738
ICD/PM, n (%)	8 (4.6)	6 (4.9)	2 (4.0)	0.650
Palpitations, n (%)	130 (75.5)	96 (78.7)	34 (69.4)	0.198
Angina, n (%)	31 (18.0)	25 (21.2)	6 (12.2)	0.176
Dyspnoea, n (%)	70 (40.6)	51 (45.1)	19 (38.8)	0.453
NYHA class ≥ 2	43 (25.0)	29 (23.7)	14 (28.0)	0.639
Syncope, n (%)	11 (12.7)	8 (6.7)	3 (6.0)	0.872
Class Ic antiarrhythmics, n (%)	40 (23.3)	27 (22.1)	13 (26.0)	0.585
Class III antiarrhythmics, n (%)	48 (27.9)	33 (27.0)	15 (30.6)	0.639
Betablocker, n (%)	95 (55.2)	69 (57.0)	26 (52.0)	0.548
Calcium channels blockers, n (%)	20 (11.6)	14 (11.5)	6 (12.0)	0.922
Antivitamin K, n (%)	5 (2.9)	4 (3.3)	1 (2.0)	0.645
NOAC, n (%)	110 (64.0)	79 (64.8)	31 (62.0)	0.733
Antiplatelet, n (%)	22 (12.8)	15 (12.3)	7 (14.0)	0.761
RAAS blockers, n (%)	60 (34.9)	46 (37.7)	14 (28.0)	0.225
Diuretics, n (%)	28 (16.3)	21 (17.2)	7 (14.0)	0.604
Recurrence in BP, n (%)	64 (37.2)	33.0 (27.0)	31.0 (62.0)	<0.001

BMI: body mass index; AF: atrial fibrillation; CAD: coronary artery disease; CVA/TIA: cerebrovascular accident/transient ischemic attack; COPD: chronic obstructive pulmonary disease; ICD/PM: implantable cardioverter defibrillator/pacemaker; NYHA: New York Heart Association; NOAC: novel oral anticoagulant; RAAS: renin—angiotensin system; BP: blanking period.

**Table 2:** Echocardiography characteristics of the study population.

	Total (n = 172)	No AF recurrence (n = 122) (71%)	AF recurrence (n = 50) (29%)	p
LVEDV, ml	102.4 ± 36.3	103.4 ± 38.8	99.8 ±29.7	0.621
LVESV, ml	47.9 ± 24.2	48.0 ± 25.7	47.6 ± 20.5	0.942
LVEF, %	54.5 ± 18.1	54.6 ± 8.3	52.6 ± 10.3	0.276
LVEF ≥ 50%, n (%)	142 (82.5)	103(84.4)	39 (78.0)	0.325
LVEF = 41–49%, n (%)	20 (11.6)	15 (12.2)	5 (10.0)	0.657
LVEF ≤ 40%, n (%)	10 (5.8)	4 (3.2)	6 (1.2)	0.086
GLS, %	-18.0 ± 3.7	-18.2 ± 3.5	-17.4 ± 4.1	0.301
E/A ratio	1.2 ± 0.4	1.2 ± 0.4	1.2 ± 0.5	0.848
E/e’ratio	7.7 ± 3.0	7.9 ± 2.8	7.2 ± 3.6	0.231
TR max PG, mmHg	25.0 ± 7.5	24.3 ± 7.0	26.5 ± 8.3	0.186
IVC, cm	1.3 ± 0.6	1.2 ± 0.7	1.3 ± 0.4	0.378
Mitral regurgitation, n (%)	127 (73.8)	85 (70.2)	42 (84.0)	0.061
Aortic regurgitation, n (%)	44 (25.5)	29 (24.2)	15 (30.0)	0.429
Tricuspid regurgitation, n (%)	127 (73.8)	88 (72.7)	39 (78.0)	0.473
Valvular stenosis, n (%)	0 (0.0)	0 (0.0)	0 (0.0)	-
LADI, cm/m^2^	2.1 ± 0.3	2.1 ± 0.3	2.3 ± 0.3	0.001
LAVI ml/m^2^	34.8 ± 12.0	32.9 ± 11.6	39.6 ± 11.7	0.001
Global PALS, %	24.5 ± 11.5	26.3 ± 10.8	20.0 ± 12.1	0.002
Global PACS, %	14.7 ± 15.5	15.3 ± 5.5	12.9 ± 5.4	0.038
Global conduit strain, %	13.6 ± 7.7	13.6 ± 8.0	13.7 ± 6.7	0.943

LVEDV: left ventricle end—diastolic volume; LVESV: left ventricle end—systolic volume; LVEF: left ventricle ejection fraction; GLS: global longitudinal strain; TR: tricuspid regurgitation; IVC: inferior vena cava; LADI: left atrium diameter index; LAVI: left atrium volume index; PALS: peak atrial longitudinal strain; PACS: peak atrial contraction strain.

Cryoballoon ablation procedural data is summarized in S1 Table.

### Predictors of AF recurrence

During the follow -up period of 11.7 ± 1.6 months, 50 (29%) patients had at least one episode of AF recurrence after the BP, resulting in an AF-free survival rate at 12 months of 73.1 ± 5.0%.

The optimal cut-off value of PALS for the prediction of AF recurrence was 17%, with a sensitivity of 82% and a specificity of 58%. For LAD index, sensitivity and specificity for the prediction of AF recurrence were 72% and 53%, for an optimal cut-off value of 2.36 cm/m^2^. LAV index had a sensitivity and specificity of 78% and 48% for an optimal cut-off value of 41.9 ml/m^2^.

Univariable analysis for the prediction of AF recurrence is shown in S2 Table.

**Fig 2:**
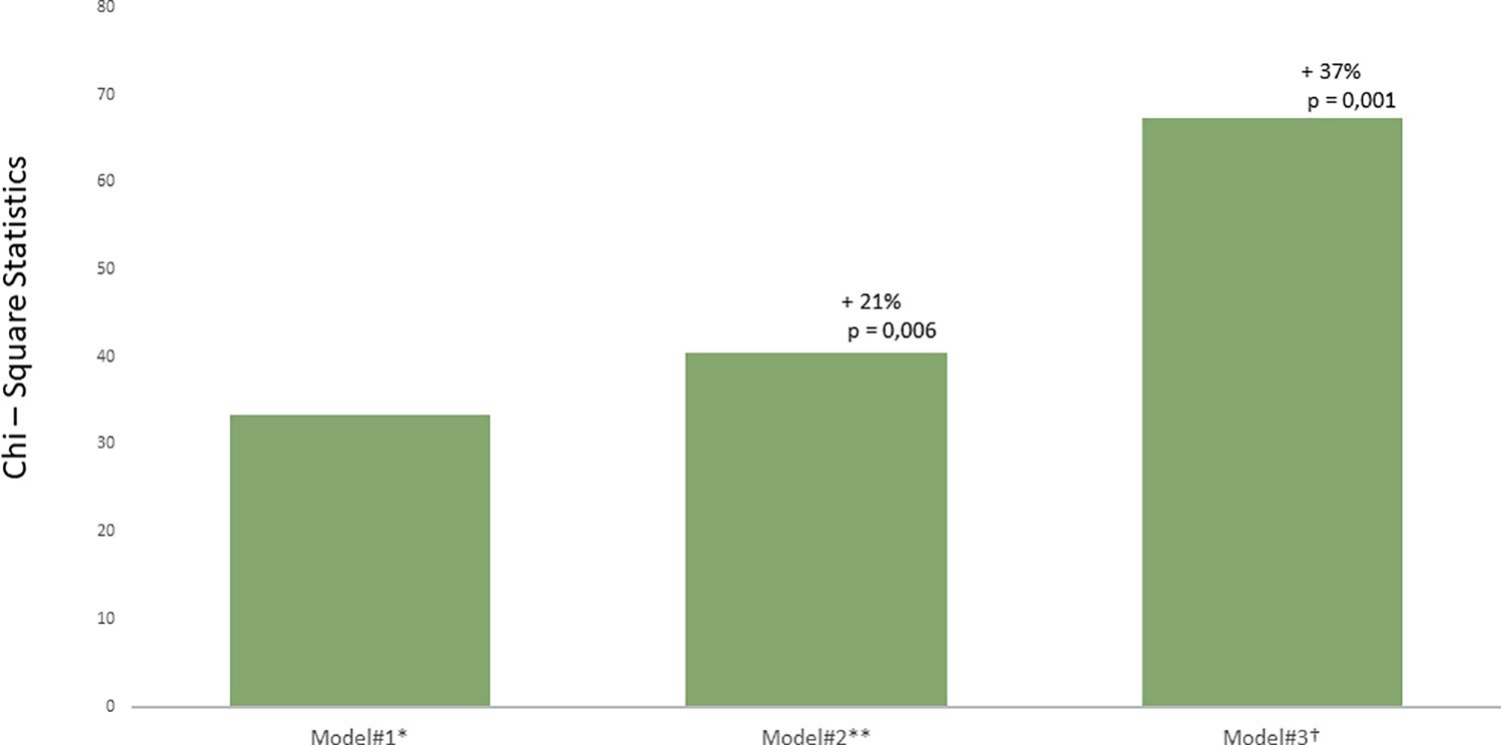
Chi—Square statistics for different models for the prediction of atrial fibrillation (AF) recurrence. Values indicate the percentages of increase in Chi—square for every model. Statistical comparison by likelihood ratio tests. *Model#1: persistent AF, recurrence during the blanking period, left atrium diameter index ≥ 2.36 cm/m^2^; global Chi—square = 33.4. **Model#2: Model#1* + left atrium volume index ≥ 41.9 ml/m^2^, 21% increase in Chi—square. †Model#3: Model#2* + peak atrial longitudinal strain ≤ 17%, 37% increase in Chi—square.

Table 3 shows the multivariable models for the prediction of AF recurrence. PALS ≤ 17% had the highest incremental predictive value for AF recurrence (HR = 9.45, 95% CI: 3.17–28.13, chi—square to improve: 37% increase, p < 0.001) (Fig 2).

**Table 3:** Multivariable analysis for the prediction of AF recurrence after the blanking period.

Multivariable analysis								
	HR	95% CI	Chi—Square	P value	Chi -square improvement (% of increase)	HR	95% CI	P value
**Model#1** *								
Persistent AF	3.2	1.2–8.4	6.4	0.014				
Recurrence during BP	5.0	2.3–1.0	18.2	0.001				
LADI ≥ 2.36 cm/m^2^	3.0	1.3–7.0	4.06	0.006				
**Model#2** **								
LAVI ≥ 41.9 ml/m^2^					+21%	3.2	1.4–7.0	0.006
**Model#3** †								
PALS ≤ 17%					+37%	9.4	3.1–28.1	<0.001

AF: atrial fibrillation; BP: blanking period, LADI: left atrium diameter index; LAVI: left atrium volume index; HR: hazard ratio; CI: confidence interval; PALS: peak atrial longitudinal strain.

*Global Chi—square of Model#1 = 33.4

**Model#1 + LAVI ≥ 41.9 ml/m^2^

† Model#2 + PALS ≤ 17%.

C-Statistic **Model#1** = 0.762; C–Statistic Model#2** = 0.782; C-Statistic Model**#3† =** 0.807.

**Fig 3:**
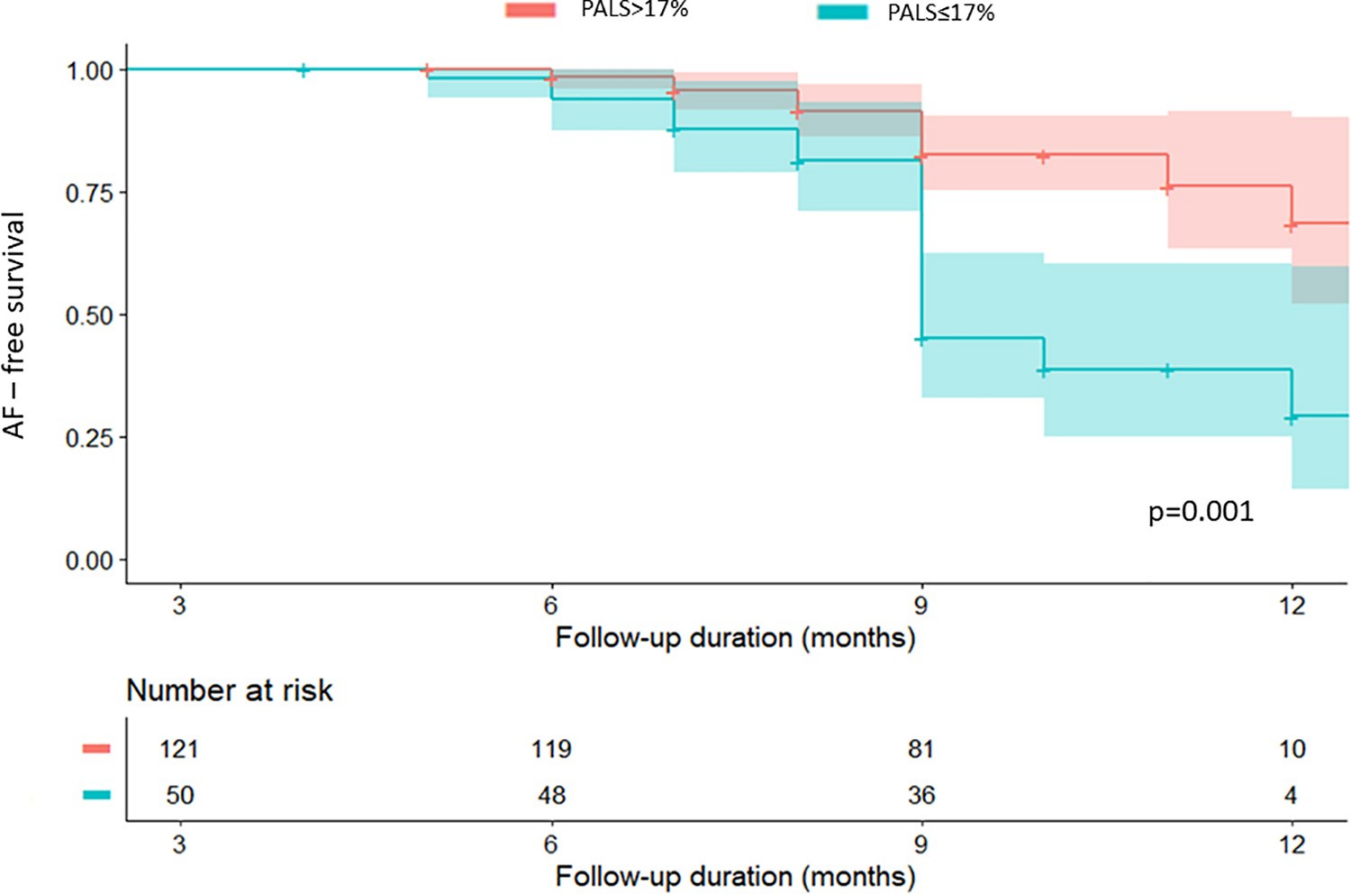
Kaplan—Meier AF—Free survival curve stratified by the optimal cut-off value of peak atrial longitudinal strain (PALS).

Patients with decreased PALS had reduced AF-free survival (29.0 ± 10.6% for PALS ≤ 17% versus 76.1 ± 7.0% for PALS > 17%, log–rank = 0.001) (Fig 3).

### PALS in patients with normal LA size

One hundred nineteen patients (69.1%) had a normal LAD index. In this category of patients, AF recurrence occurred in 24 patients (20.2%), resulting in an AF—free survival rate at 12 months of 76.0 ± 6.0%. PALS was significantly different between patients with and without AF recurrence (22.2 ± 11.6% versus 27.3 ± 9.8%, p = 0.035). For an optimal cutoff value of 17%, PALS had a sensitivity of 87% and a specificity of 49% for the prediction of AF recurrence. The univariable and multivariable Cox regression analysis for this category of patients is shown in S3 and S4 Tables, respectively.

### Intra- and interobserver reproducibility of LA measurements

Intraobserver variability was good for all analyzed parameters: for LAD ICC 0.972 (95% CI 0.899–0.985), for LAV ICC 0.964 (95% CI 0.860–0.980), and for PALS ICC 0.950 (95% CI 0.835–0.982), respectively. Interobserver variability was good for all analyzed parameters: for LAD ICC 0.906 (95% CI 0.750–0.940), for LAV ICC 0.907 (95% CI 0.810–0.950), and for PALS ICC 0.893 (95% CI 0.719–0.960).

## Discussion

In the present study, PALS provided an incremental predictive value for AF recurrence after CBA, compared to LAD index and LAV index, respectively. Additionally, PALS independently predicted AF recurrence in patients with a normal LA size.

AF recurrence might be associated with increased morbidity and mortality, due to silent AF episodes, which are difficult to detect and possibly lead to severe complications such as cerebrovascular embolic events, heart failure or death [17, 18].

Recent reports have shown a recurrence rate of AF after CBA up to 20–30%, which is in line with the results of the present study, where AF recurrence rate was 29% [19]. In order to increase the success rate, re-do ablation might be performed, but repeated procedures are usually more extensive and prone to higher risks of complications [20, 21]. Taking into consideration the morbidity attributed to re-do procedures, potential predictors of AF recurrence should be evaluated before the index ablation for a better risk stratification and to allow an individualized therapeutic strategy [22].

Previous reports have shown that early recurrences of AF during the first three months post-ablation independently predict AF recurrence after the BP, which is similar to our findings [23]. In the present study, higher rates of AF recurrence were found in patients with persistent AF, similar to the results of a report conducted by Irfan et al., which showed that persistent AF was a strong predictor of AF recurrence during a follow—up period of one year [24]. Although the presence of persistent AF per se indicates a higher risk of AF recurrence after CBA, current recommendations propose an extensive evaluation of modifiable and unmodifiable risk factors for AF recurrence in this subpopulation, for an individualized and more balanced therapeutic pathway. Moreover, the association of LA anatomy and functional parameters in addition to the classical evaluation of patients with persistent AF might provide a better risk stratification, hence an optimized management [1]. A growing body of evidence reported LA remodeling assessed by the LA transversal diameter and LAV as an independent predictor of AF recurrence [25, 26]. However, atrial remodeling in AF is a complex process that is still poorly understood. Besides the structural remodeling defined as atrial dilation, in AF the LA undergoes a maladaptive process leading to LA fibrosis and dysfunction [27]. LA function can be easily evaluated using several echocardiographic methods, including the volumetric approach or strain analysis (8).

The association between LA strain and AF recurrence was previously investigated in patients undergoing radiofrequency (RF) ablation [11, 28]. Morris et al. showed that LA strain independently predicted AF recurrence after RF ablation with an optimal cut-off value of PALS of 18.8% [29]. In patients undergoing CBA, the role of LA function in the prediction of AF recurrence has not yet been thoroughly investigated. In the present study, for an optimal cutoff value of 17%, PALS was associated with a high sensitivity for the prediction of AF recurrence. The advantage of a highly sensitive test is that it enables the identification of patients at risk to develop AF recurrence earlier and subsequently it is likely to improve the therapeutic strategy. These results are in line with a prior report from Koca et al., where LA reservoir strain independently predicted AF recurrence after CBA in patients with paroxysmal AF, with a cutoff value of 18% [30]. However, in the previously mentioned report, the additional predictive value of PALS for the prediction of AF recurrence was not compared to classical parameters, such as persistent AF, recurrence in BP or LAD [30]. Moreover, the authors did not evaluate the subgroup of patients with a normal LA size.

We observed that PALS had a higher predictive value than LA size for AF recurrence. This finding supports the hypothesis that speckle-tracking echocardiography may represent an important tool for the detection of early LA structural abnormalities such as fibrosis and consequent impairment of LA mechanics even in the presence of normal LA size [9].

A third of the patients with a normal LA size assessed by LAD had decreased PALS. In this category of patients, PALS also predicted AF recurrence. A similar finding was noted in a publication by Mondillo et al., who showed that in patients with cardiac risk factors such as diabetes or hypertension, LA dysfunction might be present even when LA size is normal [31].

These findings emphasize the potential use of LA strain for the evaluation of patients with atrial fibrillation undergoing CBA, in addition to classical clinical and echocardiographic parameters, for a better risk stratification and patient selection. Moreover, the assessment of LA strain might play an important role in detecting subtle LA dysfunction in patients with normal LA size, who might have an increased risk of AF recurrence.

Patients with decreased PALS and increased risk of AF recurrence could benefit from another type of index ablation technique, such as radiofrequency ablation with three—dimensional electroanatomical and voltage mapping. Moreover, PALS could play a role in the decision regarding the period of antiarrhythmic treatment after ablation and the frequency of follow -up in these patients.

### Study limitations

This is a single center study with a relatively small sample size. However, to ensure adequate statistical power, we performed a post—hoc power analysis using G—Power. The post—hoc analysis yielded a statistical power of 0.96 (alpha error 0.05), which exceeds the typically desired power level of 0.80. Nevertheless, larger multicentric prospective studies are warranted to confirm our findings. Patients did not have a continuous monitoring device during follow—up, therefore asymptomatic AF episodes might have occurred unnoticed. Further prospective studies with continuous monitoring during the follow—up period should be conducted to avoid undiagnosed episodes of AF recurrence.

Moreover, studies with a follow—up period beyond 1 year should be performed. LA strain was measured on a software designed for left ventricle strain analysis and applied to the other cardiac chambers. However, this software is the most widely used in clinical practice and for research purposes for LA strain measurements. Persistent AF was present in only 16.3% of the study population, therefore the results cannot be extrapolated to this subgroup of patients. The majority of subjects in this study had a preserved left ventricle ejection fraction. However, AF recurrence rate might be different in patients with preserved versus mid-range or reduced ejection fraction therefore further studies in these subpopulations should be carried out.

## Conclusion

This study showed that LA function assessed by PALS provided an additional predictive value for AF recurrence after CBA over LA enlargement. In patients with non—dilated LA, PALS also predicted AF recurrence. These findings emphasize the added value of LA strain, suggesting that it should be implemented in the systematic evaluation of AF patients before CBA.

## Supporting information

S1 Table Procedural data. (DOCX)

S2 Table Univariable analysis for the prediction of atrial fibrillation recurrence. (DOCX)

S3 Table Univariable analysis for the prediction of atrial fibrillation recurrence in patients with a normal LAD index (≤ 2.3 cm^2^/^2^m^2^). (DOCX)

S4 Table Multivariable analysis for AF recurrence prediction in patients with normal LAD index (≤ 2.3 cm^2^/^2^m^2^). (DOCX)
