# Supplementary material for: Incremental value of left atrial strain to predict atrial fibrillation recurrence after cryoballoon ablation
Source: PLoS One. 2021 Nov 19;16(11):e0259999. doi: 10.1371/journal.pone.0259999 (PMC8604362; doi:10.1371/journal.pone.0259999)
Supplement: S1 Table — (DOCX) [file pone.0259999.s001.docx]

S1 Table. Procedural data

|  | Total  (n = 172) | No AF recurrence  (n = 122)  (71%) | AF  recurrence  (n = 50)  (29%) | p |
| --- | --- | --- | --- | --- |
| Procedure time, min | 72.8 ± 24.9 | 70.7 ± 26.6 | 77.5 ± 20.1 | 0.129 |
| Fluroscopy time, min | 18.4 ± 10.6 | 17.1 ± 10.5 | 21.6 ± 10.5 | 0.026 |
| Freezes in LSPV | 1.2 ± 0.4 | 1.2 ± 0.4 | 1.3 ± 06 | 0.284 |
| Freezes in LIPV | 1.2 ± 0.5 | 1.2 ± 05 | 1.2 ± 0.5 | 0.641 |
| Freezes in RSPV | 1.1 ± 0.3 | 1.1 ± 0.3 | 1.1 ± 0.4 | 0.871 |
| Freezes in RIPV | 1.3 ± 0.5 | 1.3 ± 0.5 | 1.2 ± 0.4 | 0.684 |
| LSPV freeze duration, s | 28.9 ± 3.4 | 28.6 ± 3.1 | 29.8 ± 4.0 | 0.052 |
| LIPV freeze duration ,s | 29.6 ± 4.7 | 29.5 ± 4.7 | 29.8 ± 5.0 | 0.671 |
| RSPV freeze duration,s | 28.2 ± 4.3 | 28.4 ± 4.5 | 27.7 ± 3.8 | 0.370 |
| RIPV freeze duration,s | 30.3 ± 8.3 | 29.6 ± 4.5 | 32.2 ± 4.0 | 0.073 |
| Min temperature in LSPV | -51.5 ± 6.0 | -51.8 ± 5.4 | -51.0 ± 7.2 | 0.495 |
| Min temperature in LIPV | -47.4 ± 5.9 | -47.6 ± 5.6 | -47.0 ± 6.8 | 0.603 |
| Min temperature in RSPV | -51.4 ± 6.3 | -51.7 ± 5.1 | -50.8 ± 8.6 | 0.523 |
| Min temperature RIPV | -49.4 ± 5.1 | -49.6 ± 4.8 | -49.1 ± 5.7 | 0.638 |
| Pulmonary vein variants |  |  |  | 0.977 |
| - none | 118 (68.6) | 86 (72.3) | 32 (69.6) | - |
| - left common ostium | 4 (2.3) | 3 (2.5) | 1 (2.2) | - |
| - right middle pulmonary vein | 37 (21.5) | 26 (21.8) | 11 (23.9) | - |
| - right common ostium | 0 (0.0) | 0 (0.0) | 0 (0.0) | - |
| - other | 6 (3.4) | 4 (3.4) | 2 (4.3) | - |

LSPV: left superior pulmonary vein; LIPV: left inferior pulmonary vein; RSPV: right superior pulmonary vein; RIPV: right inferior pulmonary vein.
