# Supplementary material for: Incremental value of left atrial strain to predict atrial fibrillation recurrence after cryoballoon ablation
Source: PLoS One. 2021 Nov 19;16(11):e0259999. doi: 10.1371/journal.pone.0259999 (PMC8604362; doi:10.1371/journal.pone.0259999)
Supplement: S2 Table — (DOCX) [file pone.0259999.s002.docx]

S2 Table. Univariable analysis for the prediction of atrial fibrillation recurrence

| Univariable analysis | | | |
| --- | --- | --- | --- |
|  | HR | 95% CI | p |
| Age | 1.004 | 0.977 – 1.032 | 0.768 |
| Male gender | 0.945 | 0.480 – 1.859 | 0.870 |
| BMI | 1.019 | 0.952 – 1.091 | 0.591 |
| Smoking | 2.185 | 0.807 – 5.918 | 0.124 |
| Hypertension | 1.223 | 0.623 – 2.400 | 0.559 |
| Diabetes mellitus | 0.451 | 0.125 – 1.633 | 0.225 |
| Dyslipidemia | 0.723 | 0.372 – 1.408 | 0.341 |
| Persistent AF | 2.972 | 1.294 – 6.826 | 0.010 |
| Coronary artery disease | 1.383 | 0.577 – 3.316 | 0.467 |
| Heart failure | 2.690 | 0.890 – 8.130 | 0.079 |
| COPD/Asthma | 0.794 | 0.206 – 3.065 | 0.738 |
| Antiarrhythmic (class IC) | 0.809 | 0.377 – 1.735 | 0.586 |
| Antiarrhythmic (class III) | 1.190 | 0.575 – 2.462 | 0.639 |
| Reccurence in BP | 4.400 | 2.192 – 8.834 | 0.001 |
| E/A | 0.917 | 0.381 -2.207 | 0.847 |
| E/e’ | 0.930 | 0.825 -1.048 | 0.232 |
| LVEF | 0.976 | 0.935 – 1.020 | 0.276 |
| LADI | 1.019 | 1.005 - 1.034 | 0.010 |
| LADI ≥ 2.36 cm/m² | 3.806 | 1.874 - 7.730 | 0.001 |
| LAVI | 1.024 | 1.010-1.039 | 0.001 |
| LAVI ≥ 41.90 ml/m² | 3.213 | 1.506 -6.854 | 0.003 |
| PALS | 0.942 | 0.910 -0.976 | 0.001 |
| PALS ≤ 17% | 5.483 | 2.603 - 11.549 | 0.001 |
| PACS | 0.920 | 0.874 - 0.999 | 0.049 |
| PACS ≤ 16% | 2.464 | 0.967 - 6.283 | 0.059 |

BMI: body mass index; AF: atrial fibrillation recurrence; COPD: chronic obstructive pulmonary disease; BP: blanking period; LVEF: left ventricle ejection fraction; LADI: left atrium diameter index; LAVI left atrium volume index; PALS: peak atrial longitudinal strain; PACS: peak atrial contraction strain
