# Supplementary material for: Incremental value of left atrial strain to predict atrial fibrillation recurrence after cryoballoon ablation
Source: PLoS One. 2021 Nov 19;16(11):e0259999. doi: 10.1371/journal.pone.0259999 (PMC8604362; doi:10.1371/journal.pone.0259999)
Supplement: S3 Table — (DOCX) [file pone.0259999.s003.docx]

S3 Table. Univariable analysis for the prediction of atrial fibrillation recurrence in patients with a normal LAD index (≤ 2.3 cm²/²m²)

| Univariable analysis | | | |
| --- | --- | --- | --- |
|  | HR | 95% CI | p |
| Age | 0.996 | 0.960 - 1.033 | 0.996 |
| Male gender | 1.071 | 0.432 - 2.660 | 0.882 |
| BMI | 1.034 | 0.947 - 1.130 | 0.457 |
| Smoking | 2.514 | 0.671 - 9.421 | 0.171 |
| Hypertension | 1.030 | 0.411 -2.581 | 0.949 |
| Dyslipidemia | 0.793 | 0.318 - 1.979 | 0.620 |
| Persistent AF | 4.143 | 1.244 - 13.793 | 0.021 |
| Coronary artery disease | 0.625 | 0.167 - 2.335 | 0.485 |
| Heart failure | 2.543 | 0.563 - 11.491 | 0.225 |
| COPD/Asthma | 0.638 | 0.073 - 5.564 | 0.684 |
| Antiarrhythmic (class IC) | 0.800 | 0.244 - 2.618 | 0.712 |
| Antiarrhythmic (class III) | 1.196 | 0.460 - 3.114 | 0.713 |
| Recurrence in BP | 5.600 | 2.136 - 14.680 | 0.001 |
| E/A | 1.254 | 0.375 - 4.195 | 0.713 |
| E/e’ | 0.838 | 0.670 - 1.048 | 0.121 |
| LVEF | 0.992 | 0.937 - 1.050 | 0.771 |
| LAVI | 1.033 | 0.987 - 1.080 | 0.164 |
| PALS | 0.942 | 0.910 -0.976 | 0.001 |
| PALS ≤ 17% | 4.125 | 1.465 -11.612 | 0.007 |
| PACS | 1.011 | 0.905 - 1.129 | 0.849 |

BMI: body mass index; AF: atrial fibrillation recurrence; COPD: chronic obstructive pulmonary disease; BP: blanking period; LVEF: left ventricle ejection fraction; LADI: left atrium diameter index; LAVI left atrium volume index; PALS: peak atrial longitudinal strain; PACS: peak atrial contraction strain
