# Supplementary material for: Incremental value of left atrial strain to predict atrial fibrillation recurrence after cryoballoon ablation
Source: PLoS One. 2021 Nov 19;16(11):e0259999. doi: 10.1371/journal.pone.0259999 (PMC8604362; doi:10.1371/journal.pone.0259999)
Supplement: S4 Table — (DOCX) [file pone.0259999.s004.docx]

S4 Table. Multivariable analysis for AF recurrence prediction in patients with normal LAD index (≤ 2.3 cm²/²m²)

| Multivariable analysis for AF recurrence prediction in patients with  normal LAD index (≤ 2.3 cm²/m²) | | |
| --- | --- | --- |
|  | HR | p |
| Recurrence during BP | 6.86 (95% CI 2.35 -20.02) | 0.001 |
| Persistent AF | 4.15 (95% CI 1.25 - 13.80) | 0.021 |
| PALS ≤ 17% | 5.39 (95% CI 1.66 - 17.52) | 0.005 |

AF = atrial fibrillation; BP = blanking period; LAD = left atrium diameter; PALS = peak atrial longitudinal strain
